# Digital learner presence and online teaching tools: higher cognitive requirements of online learners for effective learning

**DOI:** 10.1186/s41039-017-0059-3

**Published:** 2017-09-07

**Authors:** Sue Gregory, Michelle Bannister-Tyrrell

**Affiliations:** School of Education, University of New England, Armidale, Australia

**Keywords:** Digital learner presence, 3D virtual worlds, e-learning, Metacognition, Information Communication Technology (ICT), Engagement, Community of Inquiry

## Abstract

This article explores digital learner presence in various higher education degrees in a regional institution in NSW, Australia. Several tools used for online teaching are explored through individual research projects in relation to the learner’s presence with the tool being used. It was found that a variety of online teaching tools provided student presence and were effective for learning. Blogs, discussion boards, wikis and 3D virtual worlds were used to engage students in their learning. Herewith, the authors point out those that are more successful than others.

## Introduction

The authors began their careers as academics in the higher education sector in 2006 and 2013 respectively. Gregory was in the area of Information Communication Technology (ICT) Education and Bannister-Tyrrell in Gifted and Talented Education. Both academics incorporate ICT into their students’ learning as the institution where they work, the University of New England, is an online course provider to over 80% of their students. The authors had to learn to teach online and use a variety of tools to engage their students.

This paper is based on research conducted since 2008 and the academics’ perception of how various ICTs (blogs, discussion boards, wikis and 3D virtual worlds) have had an impact in relation to teaching and students’ engagement with their learning and their digital presence. Each student’s digital presence is explored through a variety of e-learning tools used by the students through the learning resources offered. As a regional university in Australia, the University of New England (UNE) has approximately 22,000 enrolled students with 80% opting to study online (i.e. off-campus, by distance) (University of New England 2014). In the School of Education, there are usually between 4000 and 5000 students enrolled, with 88% opting to study online. On-campus students in the School of Education have been declining with less than 12% choosing to attend on-campus where they would attend lectures, workshops and tutorials (McGarry 2014). The university average is around 82% online students. The authors, who are experienced in the use of a variety of online resources as teaching and learning tools, provide research results to demonstrate that some of these tools are immersive and engaging for the students’ learning experiences. These tools have provided students with a digital learner presence.

As noted, the majority of the students enrolled at UNE are enrolled in online mode, which means that they use a learning management system (LMS) to access their teaching and learning materials. A variety of ICT resources have been provided through the LMS including instructional resources. Many of these provide links to outside resources, usually secure resources where students are required to use a username and password to enter. However, there has only been a handful of units (subjects) that offered the variety of online tools that will be discussed in this article, including a 3D virtual world for learning.

This article explores the digital learner presence of the students and provides student feedback on their perceptions of the affordances of a variety of online tools used in teaching in the School of Education, namely blogs, discussion boards, wikis and 3D virtual worlds. Learner presence in this article refers to the “learner’s online self-regulatory cognitions and behaviours” (Shea and Bidjerano 2012, p. 316).

## Online tools used in this research and how they were used

A variety of online tools utilised by undergraduate and postgraduate students were involved in this research. The tools included blogs, discussion boards, wikis and 3D virtual worlds. An explanation of each of these tools follows.

### Blogs

Blogs are an information portal but began as an online diary. They enable the sharing of information and encourage collaboration. Entries are typically ordered by date, with the most recent posting at the top. Blogs were developed in the mid 1990s (Wikipedia 2010), known as a weblog which was eventually shortened to the term “blog” (Gregory 2013). “Blogging software encourages students to thinking deeper in relation to their learning and engage in discussions” (Masters et al. 2015, online).

Blogs enable a number of metacognitive and self-regulatory processes to be practiced and enhanced. As an online diary, reflection on self-knowledge including memories, knowledge of personal theories and capabilities, self-awareness and self-understanding are encouraged. There are also opportunities for the author to reflect on their knowledge of self-systems including self-efficacy, self-concept, self-esteem and self-appraisal (Tarricone 2011). The educator can manipulate which skills they wish to encourage within the blog by carefully designing guiding questions to elicit purposeful reflection. Teachers should also be very aware of the audience and design the question accordingly. If, for example, a large group will be reading the blog, the teacher needs to ensure that students do not share personal or offensive information that will negatively impact on the students’ experience of the unit, or the audience.

Metacognitive reflection offers a powerful skill set students should be encouraged to use. These skills include “reflective thinking – purposeful reflection, higher-order reasoning, critical reflection, critical thinking and reflective judgments” (Tarricone 2011, p. 199). Once again, careful design of the task can elicit enhanced practice of any one or multiple skills. For example, higher-order reasoning requires the learner to make connections with prior knowledge and understanding in order to build new knowledge. A task could be designed asking students to explain their understandings about a topic before completing new readings and then explain how their attitudes may or may not have altered as a result of the new information.

### Discussion boards

Discussion boards are also known as bulletin boards or forums and are used to post information, ideas or questions enabling others to respond asynchronously. Whereas a chatroom is used for responding to posts straight away, a discussion board enables someone to make a considered response because they have time to think about how to respond, before posting.

All units conducted by the authors have used discussion boards to communicate with students within the university’s designated LMS. Moodle has been the LMS of choice at UNE since 2011. Information is disseminated wide scale and is relatively easy using a discussion board. Students use the discussion board to post queries and/or respond to other students. They make statements and share information and resources. Discussions are often channeled by the academic, such as through *Unit Announcements* (sent to all students via email and also posted on the discussion board); *Introductions* (where students get to know each other at the beginning of a course); *Assessment Tasks* (where questions and answers in relation to specific assessment topics can be posted); *Administration* (where students could ask administrative-type questions that were not related to an assessment task, for example, how to complete forms or find them on the UNE website); *General Discussion* (students could generally add posts here that were not related to another of the other topics, for example, they may add a YouTube video that was relevant to the unit, but not specific to assessment tasks); and the *Coffee Shop* (this was a space just for the students. Questions by the academics were not answered here and it was not monitored. This space gave students an online presence and private space with their peers).

Participation in the discussion boards was voluntary (unless there was an assessment task around the posts). Students were encouraged to ask their questions here in case they were pertinent to other students.

Careful consideration of responses on discussion boards gave students the opportunity to reflect on what information they wished to share with their online peers. Less confident students may use the opportunity to read other students’ work before committing their own responses to the board. In this way, they may choose to monitor and control clarity and accuracy of the type of information they wish to share, reflective of self-regulation. The more complex the task set by educators within discussion boards, the more likely the task would elicit critical thinking or reflection (Tarricone 2011); however, the task also needed to be age-appropriate and skill-based according to the learning cohort.

### Wiki

Wikis are asynchronous tools and enable creation, collaboration, editing, linking web sites, adding images and videos and sharing with other members of the wiki. Wikis do not require the set-up and hosting of a website, and therefore, users do not need to have knowledge of web editing. Wikis were first developed in 1995 by Ward Cunningham with WikiWikiWeb and was named after the Hawaiian word for “fast” (Godwin-Jones 2003). Wikis are an excellent format for group work and can be set up to be secure from those not enrolled in the unit of study.

### 3D virtual worlds

**Fig. 1:**
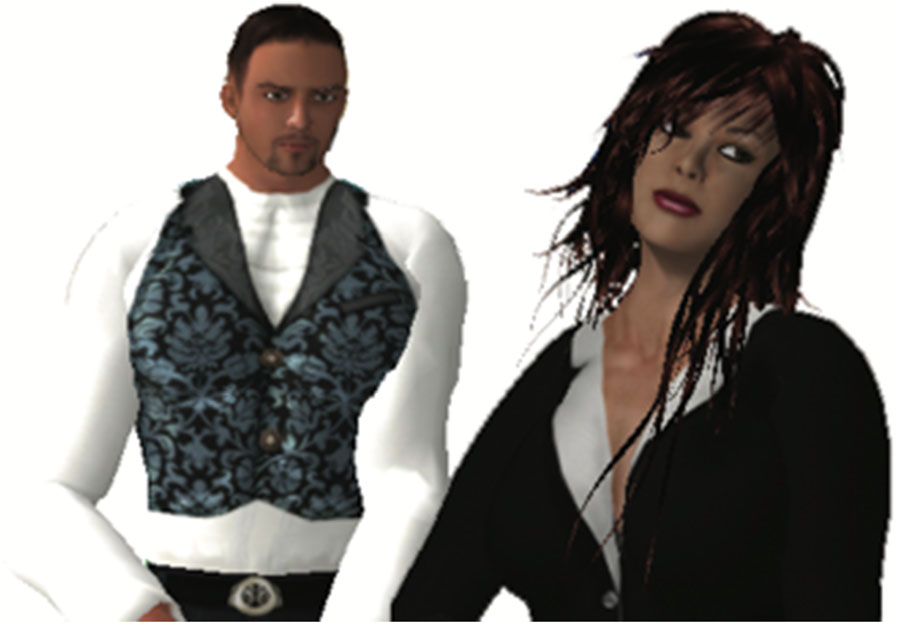
Two avatars in Second Life (academics in “real life”)

3D “virtual worlds are a low cost computer program that can simulate and/or substitute real-world activities through a person’s avatar” (Gregory 2007, online). Virtual worlds can replicate the real world in context, making it an ideal place for online teaching and learning with students. Virtual worlds embed asynchronous and synchronous resources for students to interact and engage in. Users of a virtual world require the creation of an avatar to use when entering the world and interacting with the environment and other users. Educators can set up classroom activities and tasks for their students. All users communicate via text chat or audio and are able to see each other’s avatars “live” (i.e. they are there at the same time and one is able to see other users moving around in the “world”). Discussions with other users can be one-on-one (such as through a text chat or instant message (IM)), to members of specific groups (group chat) or to anyone that is nearby, in local chat. Figure 1 provides an image of two avatar academics demonstrating how realistic they can be (these avatars were created in 2007).

As a more spontaneous activity, text chat in a virtual world tends to be more reflective of students requiring synchronous assistance from other students. This is important for students studying online, as they feel less isolated and are able to share feelings with their peers. Information within text chats in a virtual world can include clarification such as trying to find information or reaffirming the task objectives or task demands, which are reflective of metacognitive declarative and procedural knowledge.

#### Second Life (3D virtual world)

Second Life is one of many virtual worlds available for people to use. It was released to the public in 2003 by Linden Lab, the proprietors of Second Life (Jennings and Collins 2007). An environment was created where members could inhabit and build their own virtual world. Second Life has been used to emulate face-to-face models of teaching which can be used to demonstrate things that are impossible to do in real life such as conducting harmful experiments, going on virtual tours and working in collaborative teams.

**Fig. 2:**
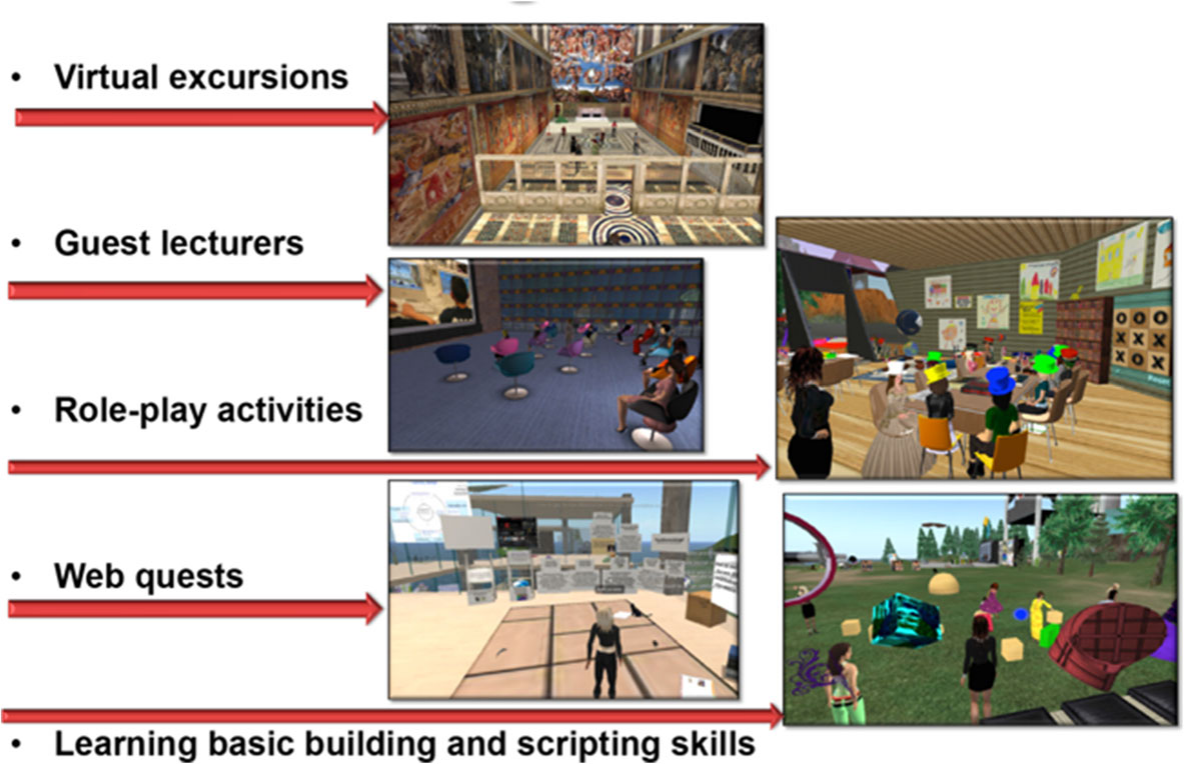
Images of activities undertaken in the virtual world

Students in the research described here have participated online to learn how to use a virtual world as a teaching and learning tool. Virtual workshops, tours, guest lectures, role-plays, web quests, learning basic building and scripting have been demonstrated to students (see Fig. 2). As can be seen by Fig. 2, there are a variety of virtual world contexts in which a student can undertake their learning. When learning in this way, students take on a digital learner presence and immerse themselves in their learning through their avatar’s persona.

Tools such as the virtual world of Second Life, where the users can create the content, if well designed by the academic, can provide the necessary structure needed for students experiencing ADHD or other executive dysfunctions that impact on working memory and processing skills (Whitaker et al. 2013). Scaffolding tasks allow students to understand the expectations of the task and assist in their development of important procedural metacognitive knowledge that will in turn assist in the development of appropriate task and strategy knowledge to complete the task and enable not only academic success but also the development of essential self-regulation skills in learners. These skills can include understanding the purpose of goal setting, monitoring progress, engagement with online tasks, activating important prior knowledge and developing reflection (Pintrich 2004; Winne and Hadwin 1998).

### Literature review

#### Constructivism

There is a growing online course industry in the higher education sector, delivering degrees to students unable to attend universities due to personal reasons. Researchers continue to search for effective teaching methods and strategies to engage online students to maximise learning opportunities and experiences. How lecturers structure online learning opportunities reflects their personal philosophies about how people learn. For many years, constructivism has been the theory of choice where students “construct knowledge” as they interact with new content, people and their own cognitive processes (Vygotsky 1978). Social constructivism focuses on knowledge building as a product of social interaction. From this perspective, collaborative learning has been highlighted as a highly effective learning tool that promotes deep learning and understanding for all students (Hattie 2009). Collaborative learning is believed to be a stimulus for cognitive development through social interaction within a group of learners (Zurita and Nussbaum 2004). Both Vygotsky (1978) and Piaget (Barrouillet 2015) promoted collaborative learning as an effective learning strategy where skills of self-regulation, negotiation of socio-conflicts and efficient communication between group members could be promoted and practiced. Neuroscience has now demonstrated how student reflection supports deeper learning, and therefore, such strategies are essential to help students connect with the world around them (Immordino-Yang 2017).

#### Andragogy and heutagogy

Undergraduate and postgraduate online learners require the development of skills that will enable them to not simply engage with online content but maximise their learning through the development of advanced skills of critical thinking and reflection that only manifest from highly developed self-knowledge and self-awareness (Gregory et al. in press). Andragogy, or self-directed learning, has been the pedagogy of choice for adult learners since the 1960s (Knowles 1984), where students focus on information related to future careers and learner experience. It is still focused on content delivery, and the learning is pre-planned by the teacher or institution. However, for postgraduate students who already have extensive experience in their careers returning to online study, a heutagogical approach (Hase and Kenyon 2000) or self-determined learning may be a more effective pedagogy to suit their learning needs. A heutagogical approach involves higher-order challenges and thinking where the student must be at the centre of their learning experiences in order to meet their professional needs and interests. Heutagogy focuses on “developing people who can cope with a rapidly changing world, a flexible workplace and uncertainty ... be proactive rather than simply reactive in their thinking, and who can be more involved citizens” (Hase and Kenyon 2000, p. 6).

#### Community of Inquiry

**Fig. 3:**
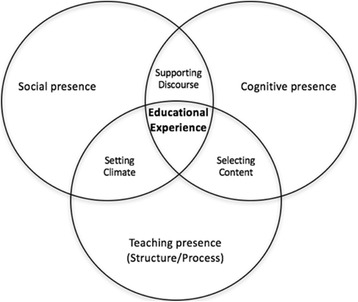
Community of Inquiry framework (Garrison et al. 2000)

In 2000, Garrison, Anderson and Archer proposed a conceptual framework integrating online collaborative learning within the Community of Inquiry. While Community of Inquiry (CoI) is not a new concept in education (Dewey 1902), these authors developed a framework specifically suited for online learning. The model highlights three over-arching elements including cognitive presence, social presence and teaching presence (see Fig. 3). Cognitive presence involves the construction of knowledge and meaning through collaboration and communication. “Cognitive presence is a vital element in critical thinking, a process and outcome that is frequently presented as the ostensible goal of all higher education” (Garrison et al. 2000, p. 89). Social presence in the CoI is the projection of students as “real people”, which again is required for authentic learning and facilitating both cognitive presence and critical thinking. The third element is teaching presence, which involves the “design of the educational experience” and the facilitation of the learning experience “to support and enhance social and cognitive presence for the purpose of realizing educational outcomes” (p. 90). CoI fits well with both an andragogic and heutagogical approach to learning for adult learners. Figure 3 provides a visual overview of Garrison et al.’s (2000) Community of Inquiry framework.

While CoI is a framework based on practical inquiry and critical thinking, when encouraging asynchronous knowledge construction in online environments (Shea et al. 2012), there is no guarantee that student engagement will be a natural offshoot of CoI. Larreamendy-Joerns and Leinhardt (2006) warn that while online learning may be appealing, the development of an online community with reflective and self-regulated learners may or may not evolve. The authors state, in line with social constructivist theory that learning is not simply a matter of acquiring knowledge, instead:

... forging an identity and becoming a member of a community of practice through active participation ... (therefore) the challenge to online education is formidable. At a minimum, it requires designers to develop online environments where students can work together on problems, pose problems to each and critique each other’s solutions. (2006, p. 589)

Larreamendy-Joerns and Leinhardt (2006) also highlight the need for online learning design that enables students to develop competencies and skills through “opportunities for participatory practice ... thus from the very onset of learning, the learner engages in questioning, makes connections, draws inferences, and validates knowledge” (p. 590). Learning must be a social act as students negotiate meaning and develop understandings.

#### Digital learning presence

With this issue in mind, Shea and Bidjerano (2012) proposed an additional element for the CoI framework, “learner presence”. The authors focused on the point that individual differences in students “play an equally important role in students’ perceptions of cognitive engagement and gains” (p. 317). These authors highlight self-regulation as required to “optimize performance” (p. 317). However, we propose that the metacognitive perspective of self-awareness, understanding and regulating our cognition is what enables effective learning to take place (Tarricone 2011).

Rienties and Rivers (2014) took a step further to explore emotional presence for a successful online learning experience. In their review of over 100 research studies, they identified several positive, negative and neutral emotions that had an impact on the learners’ attitudes, behaviour and cognition. They found that “learners’ feelings affect motivation, self-regulation and academic achievement” (p. 16) and that these emotions can occur at any stage of the learning process, and as a result, learners may have differing emotions in relation to their learning.

**Fig. 4:**
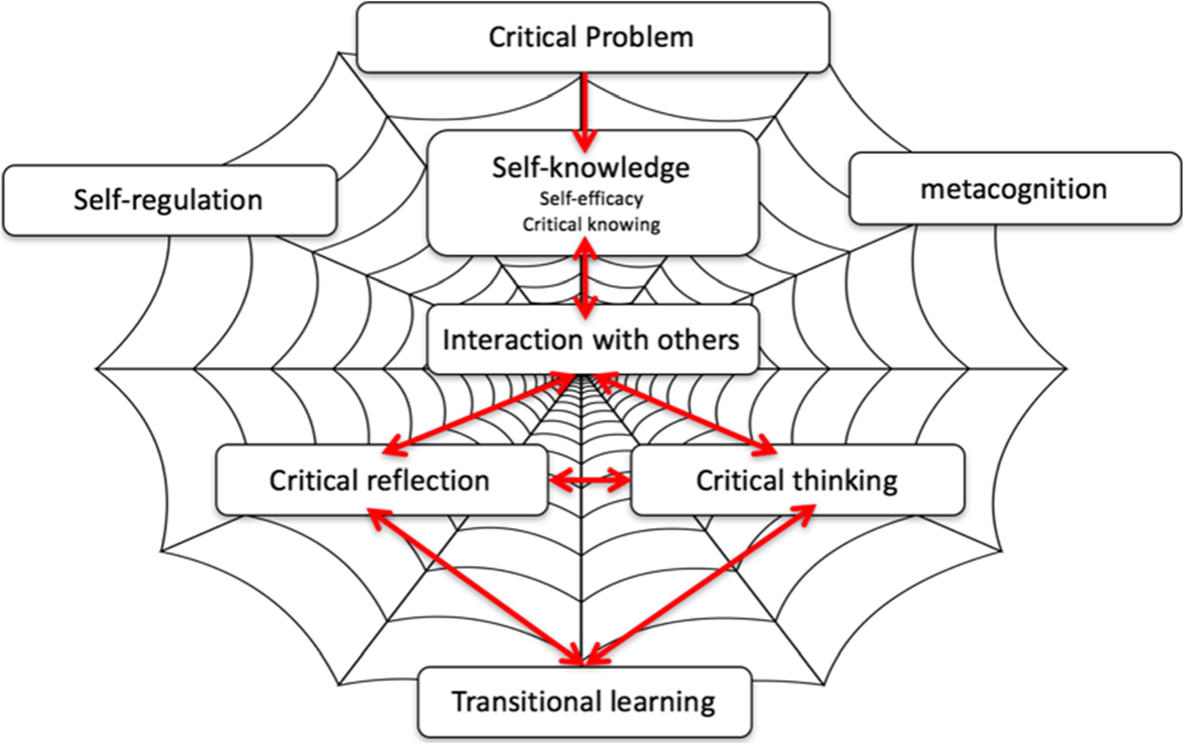
Higher-order cognitive processes (Gregory et al. in press)

Gregory et al. (in press) developed a framework demonstrating the interaction of self-knowledge, interaction with others, critical reflection, the necessary re-evaluation, reconsideration and restructuring of knowledge which leads to transitional learning (see Fig. 4). As these authors state, students need to reflect on their prior knowledge, beliefs and understandings analytically. They also need to know where to find the information and regulate their learning environment, which is essential in online interactions with others.

Figure 4 highlights how metacognition and self-regulation underpins self-knowledge, critical reflection, critical thinking and transitional learning. Higher-order cognitive processes enabled through a heutagogical approach to online study demonstrates how metacognition and self-regulation underpin and enable self-knowledge, interaction with others, critical reflection and thinking to develop from a critical problem, which in turn enables transitional learning to occur.

#### Learning online—digital presence

The research focus in this paper dealt with students learning online through the use of discussion boards, wikis and 3D virtual worlds. The authors posit that for students to be effective in their online learning, they need to have a digital online presence. Kehrwald (2008) provides an overview of how participants create an online digital presence. Firstly, they make themselves known to others through introductions, which sometimes include self-description, personal disclosure and hints of their personality. Next, they show their ongoing presence through visible activity such as posting messages to ensure others see their ongoing attendance in the online environment, their availability for communication, interaction and interpersonal relations. An individual’s social presence was seen as a cumulative result of their “demonstrations of presence but it is also affected by the strength of relations between individuals and the history of the relationship between them” (Kehrwald 2008, p. 96).

Dixson (2010) states that for effective online instruction, there also needs to be “strong instructor presence” (p. 1). Dixson (2010) also goes on to discuss several researchers who have found, through their research, that online learning can be more engaging for students (Robinson and Hulinger, as cited in Dixson 2010), have higher achievements and performance than traditional face-to-face students (Conolly, MacArthur, Stansfiled and McLesslan 2007 and Lim and Abdul-Hamid 2008, as cited in Dixson 2010) and are better with instructor interaction and communication (i.e. engagement). Dixson (2010) goes on to state that “… presence is the phenomenon that helps translate virtual activities into impressions of ‘real’ people” (p. 2). Kehrwald (2008) states that online participants experience “other participants as both real in the sense of being a real person (a human being) and present in the sense of being there in (coexisting, inhabiting) the virtual environment”. Kehrwald (2008) also found that being real and being present, in an online learning environment, are very different; however, in his research, the respondents in the online learning environment viewed their peers as real. The “respondents viewed social presence as a quality of individuals and associated it with relations between themselves and other inhabitants of the online environment as both real people and salient social actors” (p. 95).

## Methods

### Aim

The research discussed herewith explored students’ online presence, i.e. their self-regulatory cognitions and behaviours. Students from 2008 to present have participated in this research from a variety of educational degrees and contexts but all expressing their understanding and perceptions of their engagement in their learning through the use of a variety of online tools. The research aimed to provide a starting point, through the context of the student’s knowledge of their digital learner presence, and their perceptions of some of the ICTs used in their learning. A variety of research projects are presented here to provide an overview of the student’s digital learner presence in online learning. This is a longitudinal study of several research projects merged to provide an understanding of the student’s digital learner presence in relation to various ICTs and e-learning resources.

### Participants

The participants in this research were both undergraduate pre-service teachers and postgraduate education students. Postgraduate education students could only study online, whilst undergraduate pre-service teachers had the option to study either online or on-campus (face-to-face). Participation was voluntary in all the research discussed here.

### Processes

Students responded to a variety of questionnaires, mostly before and after their study sessions. They were also observed through different means (both online and face-to-face), and recording of text responses were analysed. The different types of observations were face-to-face, online posts on discussion boards, wikis and 3D virtual worlds.

### Methodology

Students were enrolled in an education degree at the University of New England. They were both undergraduate and postgraduate students and either studying online or face-to-face (on-campus) as outlined previously. An action research methodology was used, analysing both qualitative and quantitative data. Online posts, surveys, observations and student feedback were analysed to address the main research questions that emerged from the literature. These questions were centred around the following themes: student engagement and the impact on student perceptions of their learning. These themes centred around the student’s digital learner presence. All students were enrolled in an ICT unit. However, some were mandatory units, and therefore, it cannot be assumed that the students that chose to study these ICT units had prior knowledge, understanding and digital presence in any ICT tools.

## Results and Discussion

**Fig. 5:**
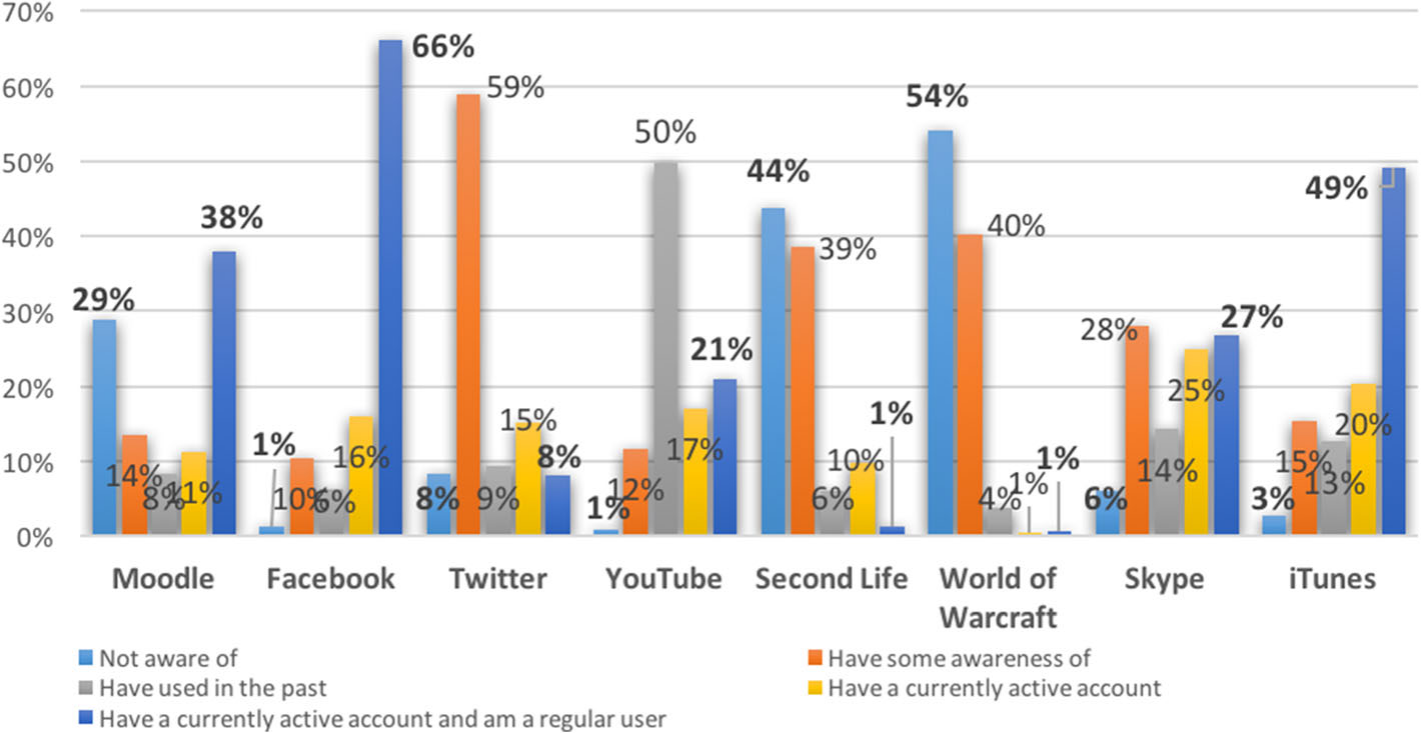
Student’s awareness of various ICTs prior to beginning their higher education studies

Reported here are the results of several research projects exploring student learner presence of various tools used in learning and teaching. Examined in more detail are blogs, discussion boards, wikis and virtual worlds. Since 2011, students have been asked to reveal what they knew about various ICTs prior to beginning their studies. Figure 5 provides an overview of what the participant students’ knowledge was of various tools prior to beginning their studies. As can be seen, Moodle, Facebook, Twitter, YouTube, Second Life, World of Warcraft, Skype, iTunes and Flickr were the ICTs that were explored. There were over 500 respondents and students ranged from pre-service teachers to postgraduate education students. Students were asked to identify if they had knowledge of these various tools and to what extent.

Moodle, the LMS that was, and still is, being used by the University, was the first ICT that students were asked if they knew prior to beginning their studies. This was important because, as has been mentioned, over 80% of the students at UNE chose to study in online mode. Therefore, an understanding of how to use the learning management system was crucial. However, it was not a requirement that students had knowledge of the LMS prior to study as they would be provided with the knowledge once enrolled. Twenty-nine percent of respondents indicated that they had no knowledge of Moodle prior to beginning their studies. Thirty-eight percent indicated that they had an account and were an active user. It could be assumed that these respondents were school leavers and had used Moodle whilst studying at school. Or, alternatively, students could have studied at another institution, or in another course at UNE, prior to commencing their studies with the authors, when asked this question. Unfortunately, the question was not asked for respondents to clarify their answer.

It is no surprise that 66% of the respondents had prior knowledge and are active users of Facebook. Only 1% had no prior knowledge of Facebook.

Again, it was not surprising that those who had used Twitter were limited, with only 1% being active users. Although Twitter is a very good resource for teachers, the respondents were not aware of this at the time of enrolling in these units. In relation to YouTube, most respondents indicated that they had either used YouTube, had an active account and/or were a regular user of YouTube, 87.8%.

The virtual world of Second Life was not known by many of the respondents, 44% indicating that they were not aware of it. Eighty-three percent indicated that they were either not aware of it or had never heard of it, and had not used it. Interestingly, World of Warcraft was included so that a comparison could be made between the two virtual worlds. Ninety-four percent of the participants either had no knowledge of this online provision or had never heard of it and therefore did not have a World of Warcraft account. This was a surprising result because this question was added so that respondents would have two virtual worlds to demonstrate their knowledge. World of Warcraft is a popular virtual world for a younger audience, and these results indicated that the respondents were more mature (which corresponds with the “typical” demographic of a UNE online student—aged around 35 years, female and working). Therefore, they may not be the “typical” person that played World of Warcraft. Second Life is a virtual world that is created by the users—all the content is made by individual users via their avatars and can be created according to one’s taste (and ICT abilities). On the other hand, World of Warcraft is highly structured, with set tasks that have to be undertaken, and the world and avatars cannot be changed. In World of Warcraft, all players join teams to progress through levels. There are no levels in Second Life, there is no game plan and individuals can exist without the need for others.

Almost one third, 27%, were regular users and account holders of Skype. This indicates that this is an ICT tool that students may use to communicate with others. Thirty-four percent of users did indicate that they either had no awareness of (6%), or some awareness of (28%), Skype.

As many students own some sort of Smart Technology, iPhone, Smartphone, iPad, Tablet or tools along these lines, it was not surprising that almost half of the respondents, 49%, had an iTunes account and were active users. This tool is used to download music or to purchase apps, ebooks, view videos, etc. However, most people were aware of iTunes or were active users, 69%. Only 3% of respondents were not aware of iTunes.

The following section discusses the tools that were explored in this paper and the research undertaken to get a sense of the learner’s digital presence through the use of these individual tools. A variety of research projects were used to provide these results. Each project is discussed briefly.

### Blogs

**Fig. 6:**
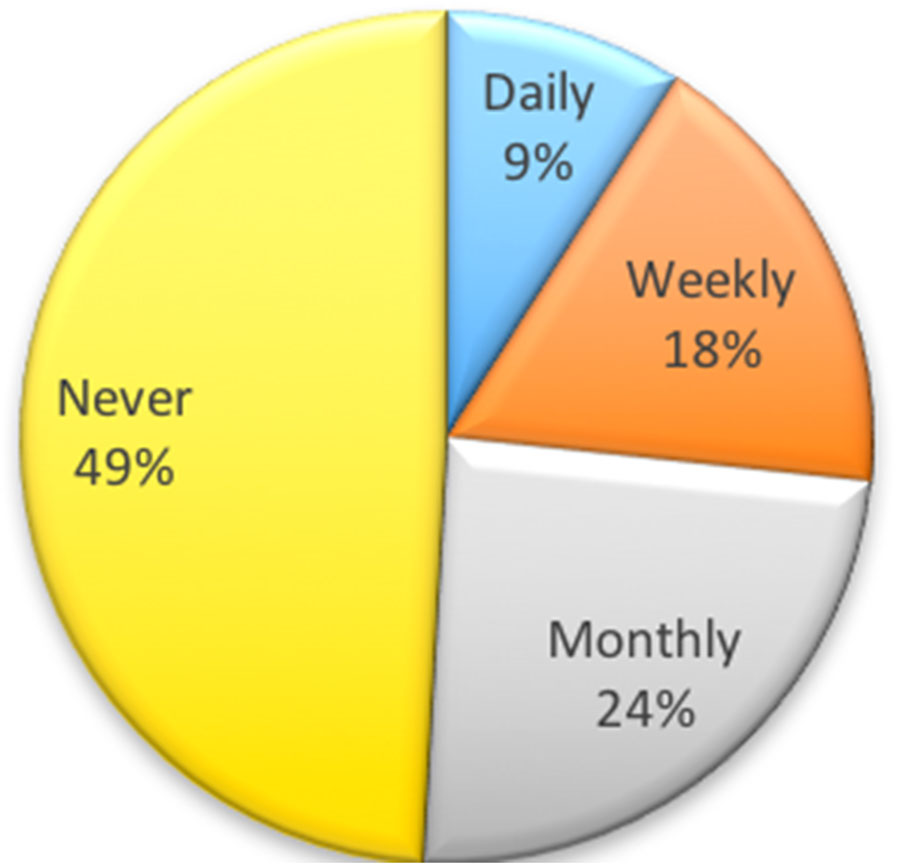
How often students used blogs

Over a 2-year period, students were asked how often they used a variety of tools. With regard to blogs, almost half of the students (49%) indicated that they did not use blogs, with only 9% using them on a daily basis. Figure 6 shows the breakdown of usage.

These responses could indicate that students were unaware of blogs or that they did not find a use for them in their online presence. They may well have used them in their studies, but the responses were for personal use, as opposed to using this tool for their studies.

### Discussion boards

**Fig. 7:**
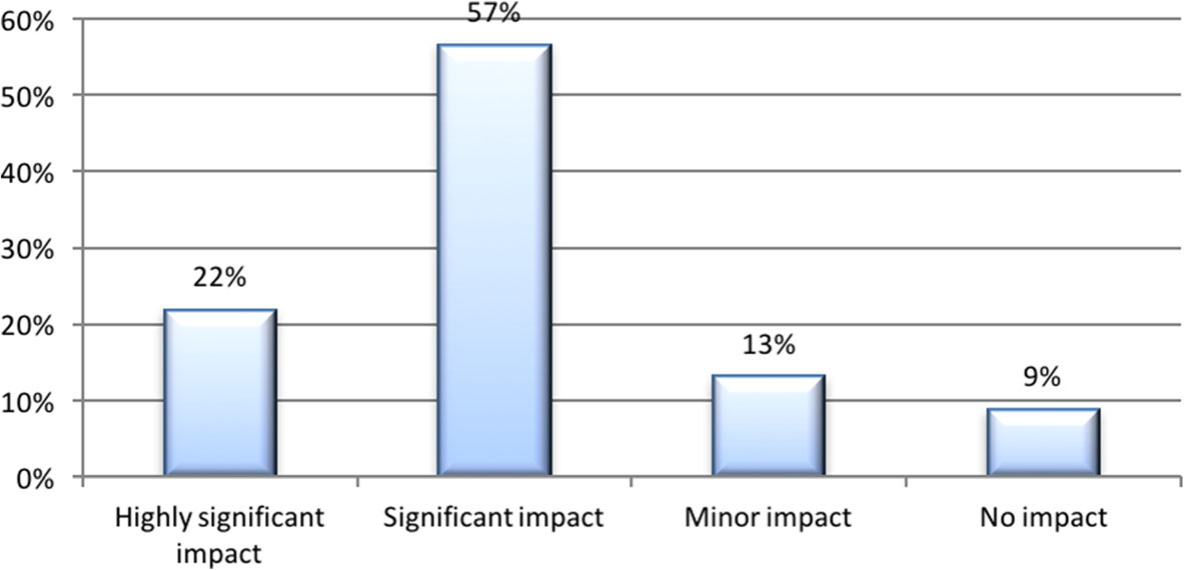
Student perceptions of their learning of concepts through discussion board conversations

Research on the use of discussion boards as a resource was conducted from 2008 to 2011 (Gregory 2015). One hundred ninety-five students responded to a questionnaire in relation to their use of various ICT resources. There were 31 (16%) males and 164 (84%) female respondents. This is typical of the gender balance of those enrolled in these education units at the University of New England. There were 64 (33%) on-campus and 131 (67%) online students who completed the questionnaire. Figure 7 indicates that the majority, 57%, felt that a discussion board had a significant impact on their learning, with 79% indicating that a discussion board had either a significant impact or a highly significant impact on their learning. Only 9% felt that the discussion board had no impact on their learning.

**Fig. 8:**
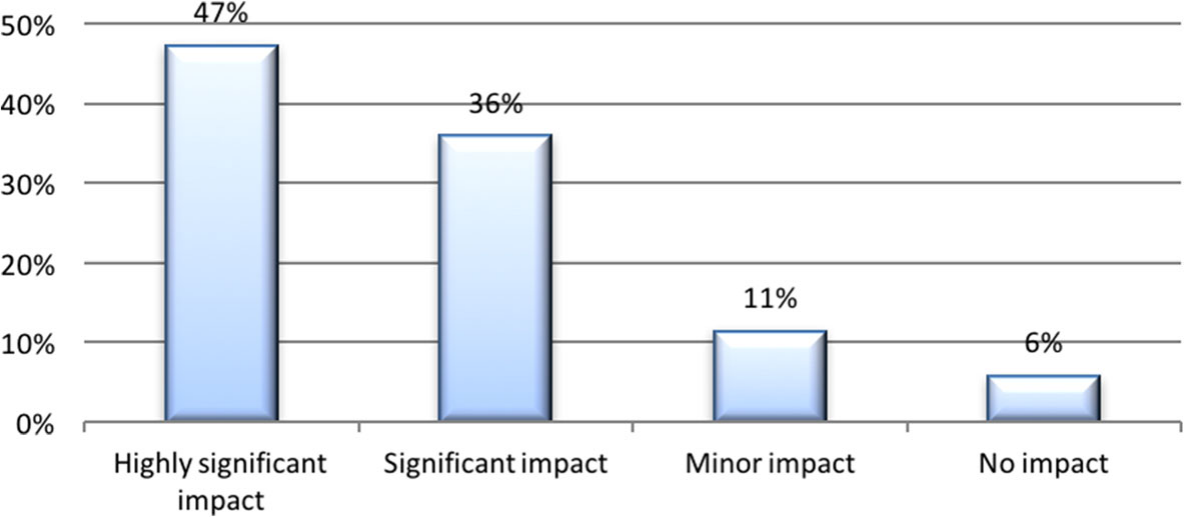
Student perceptions of the effect of discussion boards to enhance their learning

There was an overwhelming majority of students indicating that a discussion board was either highly significant, 47%, or significant, 36%, to enhance their learning, with a combined total of 83% participants. Figure 8 demonstrates how the discussion board was perceived as a very good tool to grasp concepts and enhance their learning.

The following two statements sum up how two students felt about engagement of their learning through the use of a discussion board:

As an off campus student online communication was essential. The discussions boards were the perfect way to keep in touch with other students about how they were doing and what they were doing*.*

I found the discussion board easy to use and a helpful tool to connect to the lecturer and other students. This is very useful when you are an off campus student as you don’t feel so isolated*.*

### Wikis

**Fig. 9:**
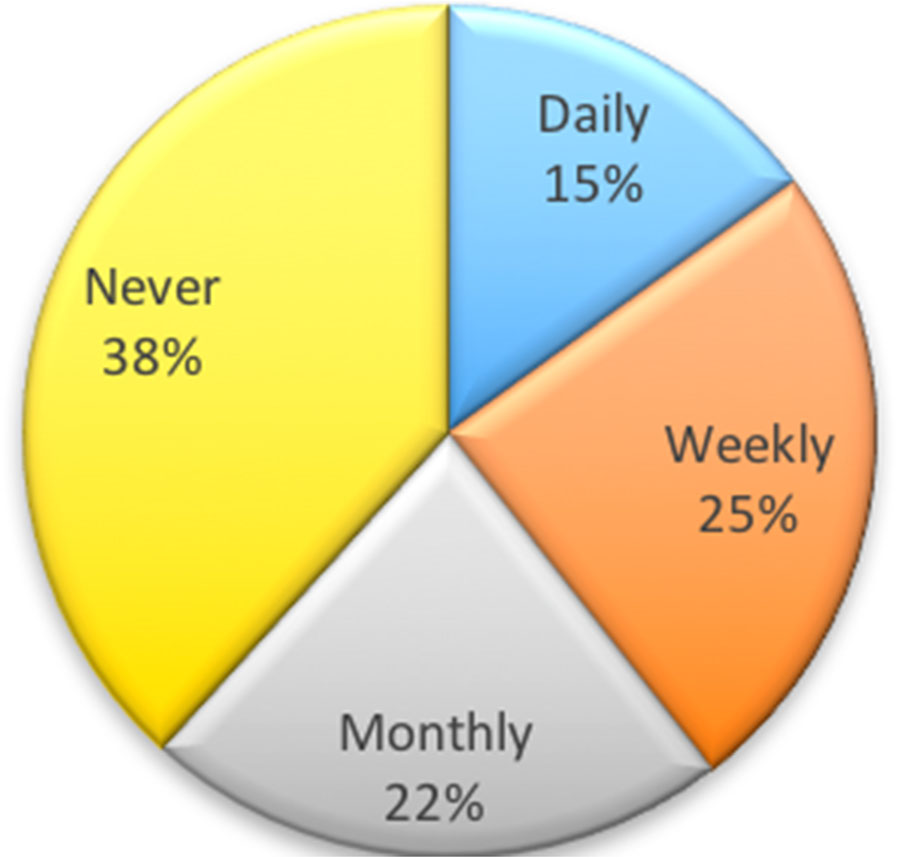
How often students used wikis

Again, over the same 2-year period where students were asked how often they used a variety of tools, students were asked about their use of wikis. Thirty-eight percent indicated that they did not use wikis; however, there were 15% using them on a daily basis. Figure 9 shows the breakdown of usage.

In all the units referred to in this research, students were required to use wikis as a teaching and learning tool. However, the results of Fig. 9 asked the question of students how often they used wikis in their own personal learning, and not required by their study.

### 3D virtual world—Second Life

**Fig. 10:**
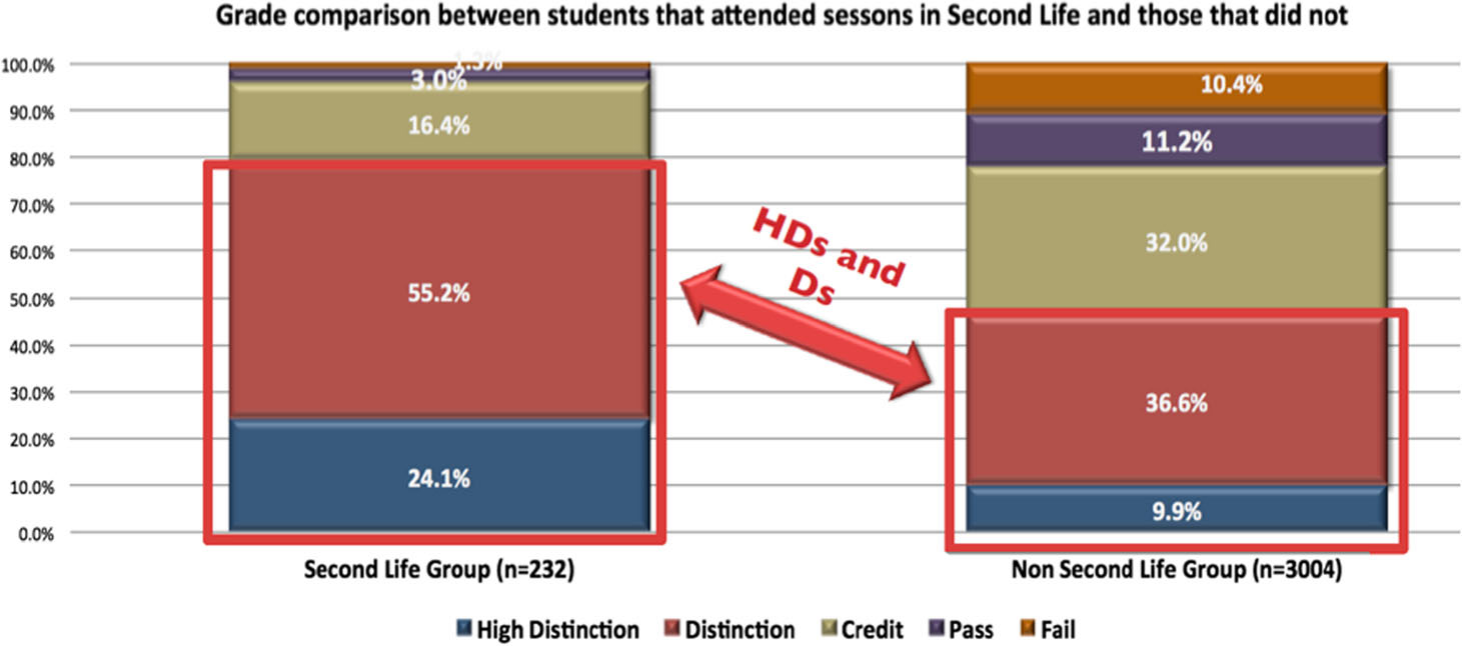
Impact of learning in a virtual world of 3576 students across a 4-year period

Since 2008, 749 pre-service teachers and postgraduate education students have participated in 3D virtual world activities in Second Life. Student participation, engagement, perception of enjoyment and grades have been analysed during this period. Over a 4-year timeframe, student grades were analysed to ascertain whether there was a difference in grades of students who participated in Second Life workshops and those who did not. Participation in these workshops was voluntary. Figure 10 shows a clear distinction in grades of those who participated in virtual world sessions and those who opted not to. There were 3236 student grades compared over the 4-year period. The Second Life participants in the left-hand column had 232 participants. The right-hand column reflected those who opted not to participate in the Second Life sessions, which included 3004 student grades. These results indicate that, on average, students who participated in the Second Life session received a higher grade than those who chose not to study using Second Life. There were 24.1% of students who received a High Distinction (85% or higher grade), of the Second Life participants, compared with 9.9% of students who did not participate in these sessions. Fifty-five percent of the Second Life participants received a Distinction grade (a grade of between 75% and 84%) compared with 36.6% of the non-Second Life participants. The Second Life participants performed at a much higher level than the other students. Of the students who participated in the Second Life group, almost 80% received a grade of High Distinction or Distinction, i.e. a grade above 75%.

There may be many inferences drawn from these results; however, the research did not explore the reasons why nor did it undertake a cross-comparison of student grades in other units of study. Importantly, the analysis was consistent across the 4 years this study ran. It is possible that the students who volunteered to participate in the virtual world sessions may be the more motivated students and the extra curricula sessions enhanced their learning. It is also possible that these students may have received these results no matter how they undertook their learning.

**Fig. 11:**
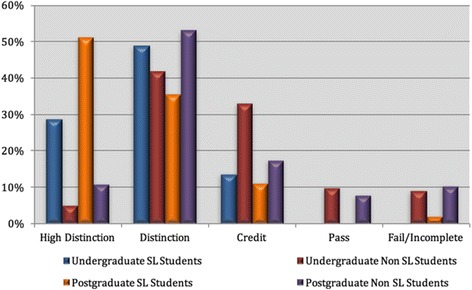
Comparison of undergraduate and postgraduate results in 2010 across seven education units: those who chose to use Second Life as part of their studies vs those who did not

Again, digital learner presence was explored through a comparison of undergraduate and postgraduate students and further compared with those students who opted to learn through the virtual world tool of Second Life and those who did not (see Fig. 11).

The dark blue and orange colours in Fig. 11 are the students who participated in the virtual world sessions (undergraduate Second Life (SL) students and postgraduate SL students). Figure 11 clearly shows that the Second Life participants received much higher grades than the non-virtual world participants.

This figure also shows that there was not a significant difference between undergraduate and postgraduate students, although the postgraduate students received more High Distinction grades than the undergraduates (who, in turn, received more Distinction grades). Figure 11 also shows the fall-off in grades from Credit to Pass to Fail/Incomplete for all students, which were significantly fewer for the Second Life participants.

## Conclusion

This article has drawn from various research projects, bringing together students’ perceptions of their digital presence from a variety of online tools, including blogs, discussion boards, wikis and 3D virtual worlds. Many of the tools were used in the students learning either as an adjunct to the learning management system or as an added extra for the student’s learning. Students gained a digital presence in many of the online tools used. Their overall feel for the tools varied with responses to discussion boards being very favourable as they felt this was a tool that could assist in their learning. For those who used blogs and wikis, they understood the context in which to use them but did not do so on many occasions of their own accord. For those students who voluntarily participated in the 3D virtual world activities, they found that the tool supported their learning and they were engaged and immersed in the content when using this tool.

What becomes apparent is that those students who have strong self-regulation and metacognitive skills are able to regulate their learning experiences. Through their interactions with others, in carefully designed collaborative learning experiences, they develop a critical consciousness, which enables them to identifying problem situations and develop solutions that address these issues. Through collaborative problem solving, these students rely on their own self-knowledge and learn to apply strategies that will enable solution finding to complex problems they encounter (Tarricone 2011). What cannot be underestimated is that for effective collaborative learning to take place through the use of technology, it requires careful design by educators and academics (Dennen and Hoadley 2013). Integrating problem-based learning into online learning, with appropriate support, will enable the development of collaborative skills that will assist their problem solving skills and self-awareness and develop communities of practice where they can develop knowledge building within this community of like-minded peers (Larreamendy-Joerns and Leinhardt 2006).

It can be concluded from this research that when a student begins their studies, they can be digitally unexposed. However, with careful scaffolding throughout their learning, they have many opportunities to gain a positive digital learner presence. Also, it was clear that some students responded both positively and negatively to different tools. They had to find the tools that suited their learning needs, whereas others found different tools suited their learning presence more appropriately. A variety of research projects were bought together to provide the opportunity for digital learner presence. The themes of the research centred around student engagement, and the impact of student perceptions of their learning, which guided the theme of this paper to present students’ digital presence through blogs, discussion boards, wikis and 3D virtual worlds.
